# All that glitters is not gold: X-ray fluorescence analysis of a fixed dental prosthesis from Colecção de Esqueletos Identificados Século XXI, Portugal (CEI/XXI)

**DOI:** 10.1007/s00414-023-03048-4

**Published:** 2023-06-22

**Authors:** Inês Oliveira-Santos, Ricardo A.M.P. Gomes, Catarina Coelho, Francisco Gil, Eugénia Cunha, Isabel Poiares Baptista, Maria Teresa Ferreira

**Affiliations:** 1 Laboratory of Forensic Anthropology, Department of Life Sciences, Centre for Functional Ecology (CFE), University of Coimbra, Calçada Martim de Freitas, 3000-456 Coimbra, Portugal; 2 Department of Life Sciences, Research Centre for Anthropology and Health (CIAS), University of Coimbra, 3000-456 Coimbra, Portugal; 3 Carrera de Antropologia, University of Concepcion, Barrio Universitario s/n, Concepcion, Chile; 4 Department of Physics and Molecular Chemical-Physics Group, Department of Chemistry, Centre for Physics of the University of Coimbra (CFisUC), University of Coimbra, Rua Larga, 3004-516 Coimbra, Portugal; 5 Institute of Legal Medicine and Forensic Sciences I. P. (INMLCF, I. P.), Lisbon, Portugal; 6 Faculty of Medicine, Center for Innovation and Research in Oral Sciences (CIROS), Institute of Periodontology, University of Coimbra, 3000-075 Coimbra, Portugal

**Keywords:** Human identification, Element analysis, Dental prosthesis, Forensic anthropology

## Abstract

Access to better health care anticipates that more medical devices can be found alongside skeletal remains. Those employed in oral rehabilitation, with available brands or batch/series, can prove useful in the identification process. A previous study in the *Colecção de Esqueletos Identificados Século XXI* described macroscopically the dental prostheses. An unusual case of a dental device with chromatic alterations demonstrated to require a more detailed analysis. The individual, a 53-year-old male, exhibited, at both arches, a fixed tooth-supported rehabilitation, with gold colouring classified initially as a gold-palladium alloy. Simultaneously, a green pigmentation deposit was observable in bone and prosthesis. This investigation aimed to verify the elemental composition of the dental prosthesis alloy. Elemental analysis was performed by X-ray fluorescence in two regions (labial surface of the prosthetic crown and the root surface of the lower right lateral incisor). Both the spectra and the qualitative results found higher levels of copper and aluminium, followed by nickel, iron, zinc, and manganese. No gold or palladium was detected. The most probable assumption is that a copper-aluminium alloy was used, as its elemental concentration corresponds to those measured in similar devices. Dental prostheses of copper-aluminium alloys have been made popular since the 1980s, particularly in the USA, Japan, and Eastern Europe. Apart from the biographical information, it was also known that the individual’s place of birth was an Eastern European country, which highlighted the usefulness of this type of information when dealing with missing people cases.

## Introduction

Forensic anthropology has significantly evolved in the last decades, thriving to adapt to the need of the practice and the challenges of today’s society [1, 2]. While identification is still the primary goal, it is expected from the forensic anthropologist to be able to gather and document as much information on context as possible to achieve scientifically sound conclusions that may be applied to the search and identification of the missing [3–5].

Nowadays, due to broader access to health care, it is anticipated that more medical devices will be found alongside skeletal remains [6]. It is conceivable that this scenario, with more clinical data available for comparison and these devices having unique characteristics such as brands or batch/series numbers, may facilitate the human identification process [7, 8].

Among the broad range of medical devices that can be found [7], those employed in oral rehabilitation are a focus of interest. Tooth loss is a widespread occurrence; thus, the presence of such devices has become more frequent across populations [9]. However, the exploitation of dental prostheses in the identification process has been hindered by an absence of brands or serial numbers, as well as a lack of systematic register on databases, and not enough research on the subject [10–12].

Seldom literature addresses the analysis of these devices [7, 13–15]. Previously, Oliveira-Santos and colleagues [10] explored the *Colecção de Esqueletos Identificados Século XXI* (21^st^ Century Identified Collection - CEI/XXI) [6] and macroscopically described the dental prostheses found in this osteological collection. No other analyses were executed to aid in the classification process. However, a specific case presenting chromatic alterations indicated the need for a more precise characterization of the metal alloy present.

The macroscopic analysis of a 53-year-old male skeleton (CEI/XXI_278; date of death: July 2011) from the CEI/XXI was previously performed in a study aimed to describe the dental prostheses found in skeletal remains and explore their application in the human identification process [10]. The devices were classified according to position, type of rehabilitation, number of units, and type of materials (please refer to [10]).

**Fig. 1:**
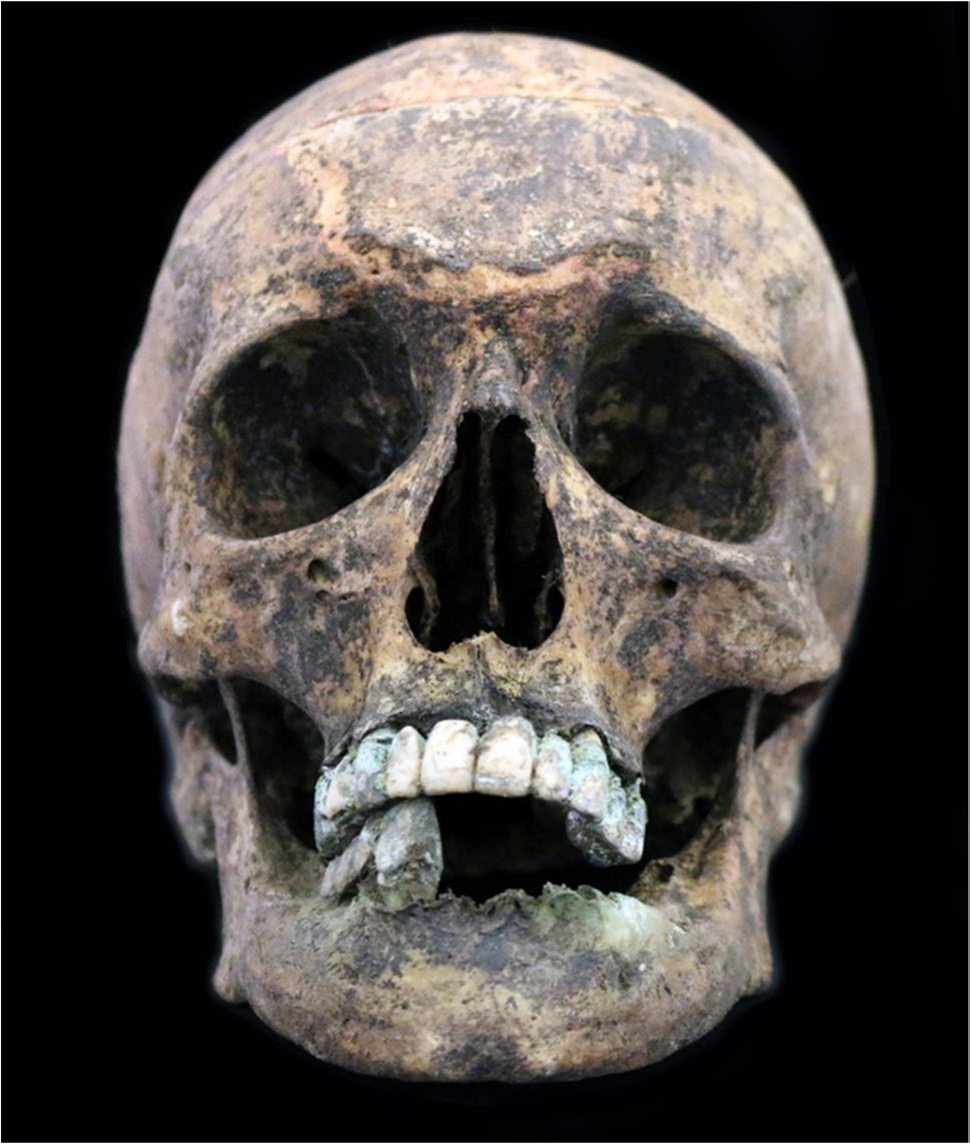
Cranium of the CEI/XXI_278 individual with fixed prostheses

The individual exhibited a fixed dental-supported rehabilitation (Fig. 1): the maxilla presented a twelve-unit bridge supported by 5 teeth (FDI 12, 15, 23, 24, and 25) and the mandible a four-unit bridge supported by 2 teeth (FDI 42 and 43), both metal-acrylic. The gold colouring of metal differentiated this case from the remaining fixed prostheses, and a classification of a gold-palladium (Au-Pd) alloy was obtained by comparison with what is applied in Portuguese dental practice nowadays.

Simultaneously, a green corrosion-like deposit was observable in both arches, over the bone and root surfaces and prosthesis. Although changes in pigmentation in skeletal remains may be the result of several factors, it cannot be excluded that the colour change may be indicative of the composition of the metal alloy present in this device [16], arousing suspicion about the initial assessment of gold-palladium.

Elemental analysis of dental devices has already been applied in forensic practice and leads to a positive identification of human skeletal remains [17–19]. Thus, this work aimed to verify the macroscopic observation by identifying the metal alloy prosthesis’s elemental composition and consequently identify the origin of the oral rehabilitation.

## Methods

Elemental analysis was conducted in a bench top fluorescence X-ray (XRF) analyser with a high-sensitivity energy dispersive Hitachi SEA6000VX HSfinder, with X-ray tube, W target, and Si multi-cathode detector, housed at the Centre of Physics of the University of Coimbra (CFisUC). X-ray fluorescence is a quantitative technique where the height of the peak for any element is directly related to the concentration of the same element. Therefore, the volume fraction of a certain element can be determined by knowing its X-ray fluorescence intensity [20].

Considering the macroscopic features of the prosthesis, the chemical analysis focused on elements that may be employed in metal alloys, such as aluminium (Al), manganese (Mn), iron (Fe), nickel (Ni), copper (Cu), zinc (Zn), silver (Ag), gold (Au), and palladium (Pd). Calcium (Ca) and phosphorus (P) were also included as they are major components of bone and dental tissues [21].

**Fig. 2:**
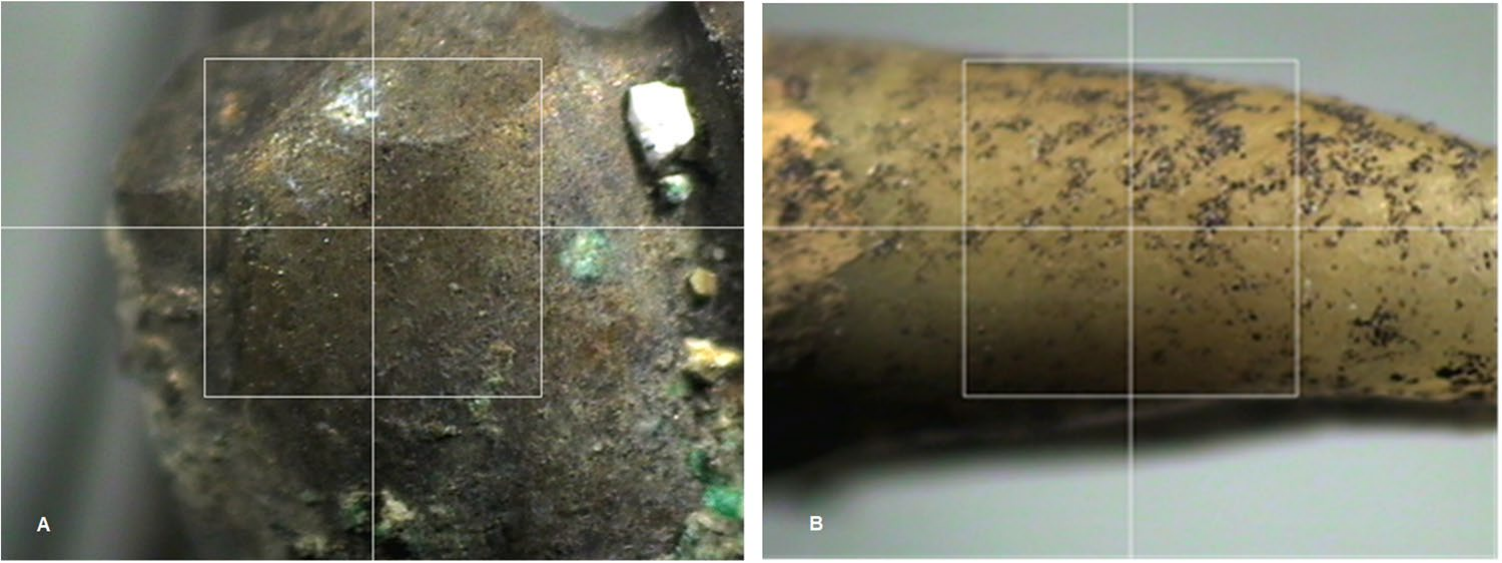
Sample view of the inferior prosthesis (**A**) and of the root of the lower right lateral incisor (**B**)

The samples were measured in two regions of the lower lateral incisor: the labial surface of the crown (Fig. 2A) and the root surface supporting the prosthesis (Fig. 2B).

## Results

**Fig. 3:**
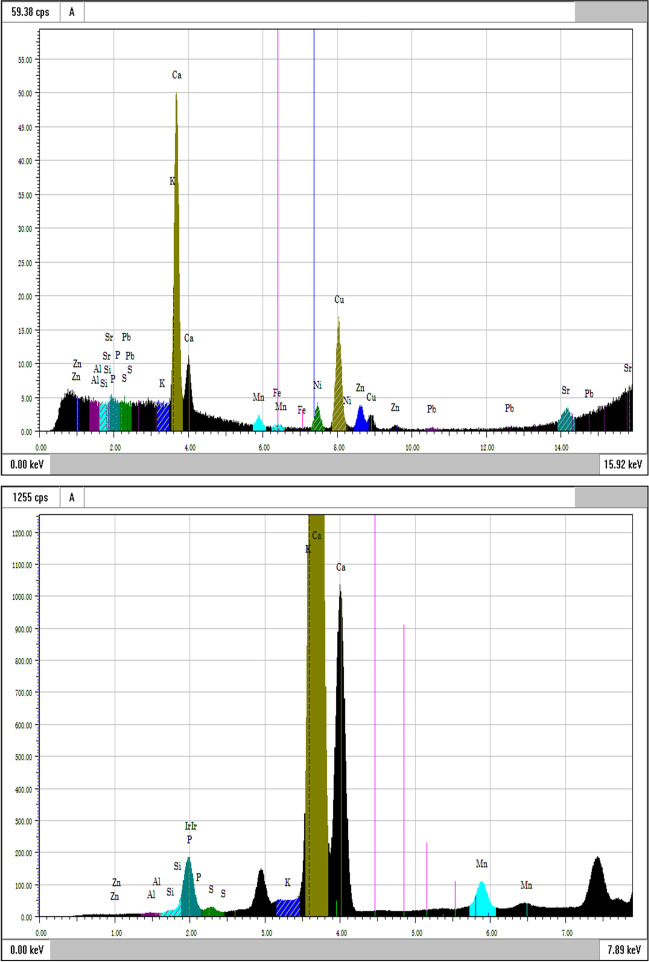
XRF spectra of the prosthesis region at high (upper) and low (bottom) X-ray energy

**Fig. 4:**
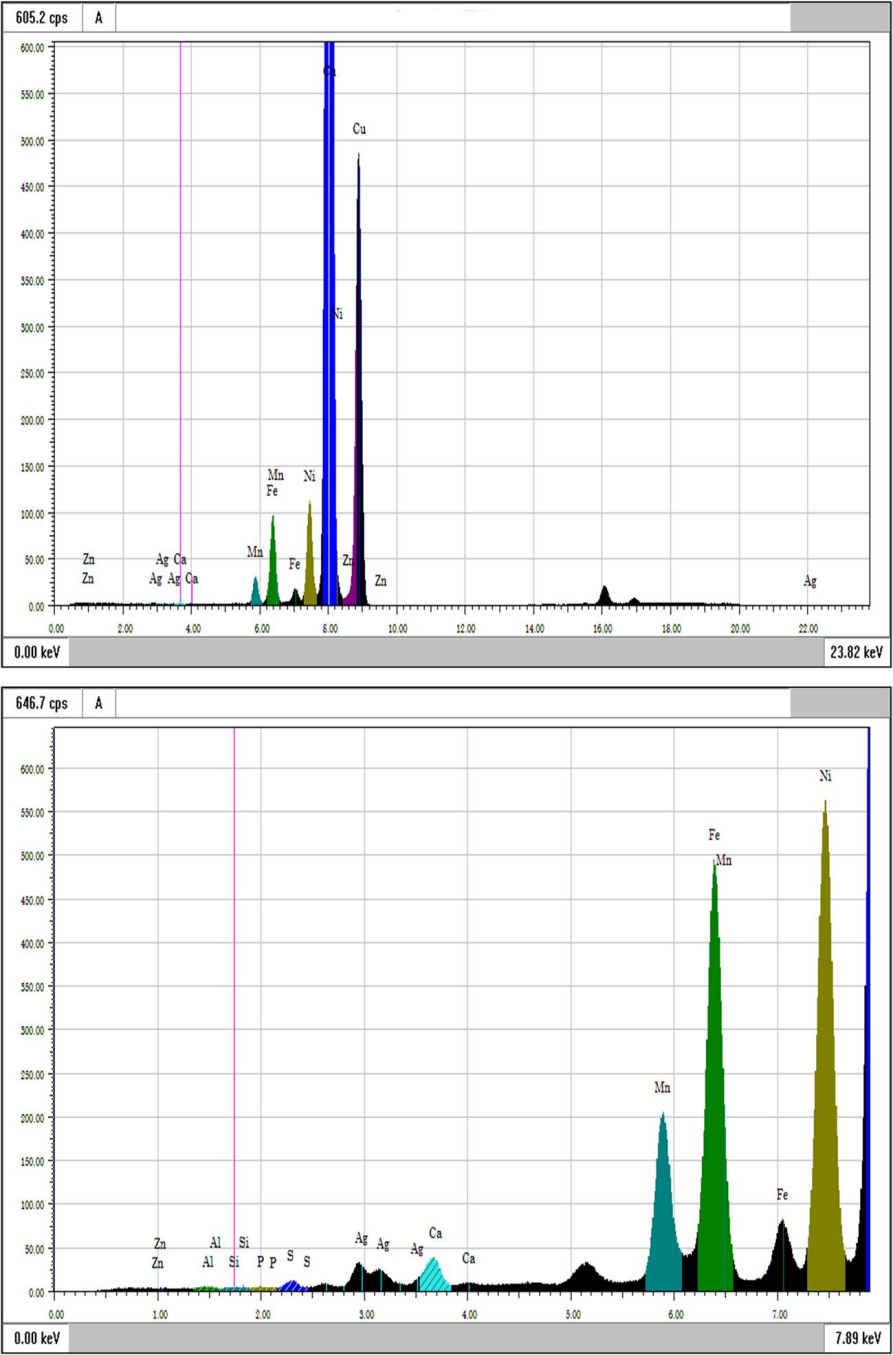
XRF spectra of the root region at high (upper) and low (bottom) X-ray energy

The qualitative results of the spectrum analysis are presented in Figs. 3 and 4. In those, high and low energy spectra are represented. The quantitative analysis was reported for the prosthesis sample and is available in Table 1.

**Table 1:** Elemental concentrations and standard deviation, obtained from the X-ray fluorescence analysis of the prosthesis region

Elements	Concentration (g/kg)	Standard deviation
Al	178.32	39.01
P	3.63	1.99
Ca	7.00	6.80
Mn	10.21	0.16
Fe	19.97	0.18
Ni	27.39	0.21
Cu	725.16	0.99
Zn	3.91	0.13
Ag	0.03	0.01

The qualitative analysis made it possible to assess that Au and Pd were not present in the device, at least not in detectable quantities.

Regarding the elements found, in the prosthesis spectrum (Fig. 3) and in the quantitative analysis (Table 1), higher levels of Cu and Al, followed by Ni, Fe, Zn, and Mn, were observed.

The root sample was explored in an attempt to assess if any elemental exchanges occurred between the device and surrounding tissues. As expected, high expressions of Ca and P were evident. Nonetheless, elements present in the alloy were also found in the root sample, namely Ni, Fe, and Mn.

## Discussion

While the macroscopic observation of the prosthesis was crucial, the alterations observed in the individual under study motivated the need for a more thorough analysis. Thus, the aim to accurately identify the metal composition of oral rehabilitation used was explored with the aid of a chemical analysis tool.

The green pigmentation observed in both prostheses and bone could, as mentioned above, has several origins. The colour change in bone is well documented, providing a good source of information for the reconstruction of the postdepositional context [16]. Although the most common causes for chromatic changes are the soil, the presence of organic materials, and/or the presence of metals, both intrinsic and extrinsic factors may affect bone, resulting in a multitude of colours [16, 22]. Thus, at first, it could not be excluded that the green pigmentation observed in this individual might have been caused by external agents. Furthermore, it is documented that skeletonized remains from cemetery contexts frequently exhibit localized stains due to the proximity to corroding mineral sources [23]. Since the skeletons from the CEI/XXI are from a cemetery context, this could have been the cause of the green colouring observed. However, this hypothesis was unlikely seeing that the different pigmentation was limited to the area of the dental prostheses and mandible, and no other area of the skeleton presented such alterations.

Focusing on the area under scrutiny, one plausible cause was in fact the presence of the metal alloy. The preliminary assessment of the alloy was a gold and palladium composition, due to the gold tone of the device. These two elements are documented to be highly resistant to corrosion, and their biocompatibility makes them a good option for oral rehabilitations [24–26]. When observing the results of the elemental analyses, there was no evidence of these two elements. It was possible that the colour change could be a consequence of the corrosion of other metals used in the device. In fact, contact with copper or copper alloy is generally associated with green and greenish blue tones [27]. In the prosthesis analysed, high concentrations of copper, aluminium, nickel, and iron were present. Hence, it was possible to assess that the hypothesis of the pigmentation being caused by corrosion of the metal alloy is the most plausible and that the presence of the green colour was an important indicator for the correct identification of the materials comprising these prostheses.

As well as the colour, the initial assessment of the alloy was also sustained by the current trends in the Portuguese dental practice reported by the dentists consulted throughout the research. However, considering the elements that comprised that part of the prosthesis in reality, the most probable assumption is that they correspond to a copper-aluminium alloy [28]. López-Alías and colleagues [29] assessed the elemental composition (X-ray diffraction) of two copper-aluminium alloys used in common dental practices (Orcast® - Madespa S.A., Spain and NPG® - Aalba Dent Inc., Fairfield, CA, USA). Levels of Al (87–106 g/kg), Mn (15 g/kg), Fe (15–21 g/kg), Ni (36–46 g/kg), and Cu (809–830 g/kg) were similar to those found in the current research, thus corroborating the identification of the alloy as copper-aluminium.

Dental prostheses made from copper-aluminium alloys have been used in various countries, particularly since the 1980s [28]. Their popularity was mainly due to their visual similarity to gold-rich alloys [30] while being less expensive as it is not comprised of noble metals [31, 32]. They are usually alloyed with minor additions of modifying elements such as Ni, Fe, and Mn [33]. However, its application became less frequent as it was demonstrated that, with time, pitting and corrosion occurred [34]. In fact, copper-based alloys corroded extensively in most laboratory tests when compared to other dental alloys, making the Au-Pd compound much more attractive [31]. In addition, colourimetric analysis has demonstrated that copper-aluminium alloys, when in contact with saliva, tend to become more yellow and slightly greener. In cases of extreme corrosion, green colours are expected to emerge abundantly [30], which explains the evident pigmentation in this case (Fig. 1).

Furthermore, concerns about its potential toxicity increased as the release of copper and aluminium ions can lead to adverse reactions, including inflammation and hypersensitivity, reasons why several stakeholders advise against its use [29].

Upon further scrutiny, it is evident that these results agree with the biographic data of the individual. Apart from the biographical information of sex and age at death, it was also available the individual’s place of birth. This 53-year-old male was born in an Eastern European country, which becomes more noteworthy when crossing this data with the reported information that copper-aluminium alloys are more usual in markets other than Portuguese, such as the USA, Japan, Brazil, and Eastern European countries [28].

It is important to remark on the usefulness of this type of information when dealing with missing people cases. While it must be cautiously used, this data may aid to reconstruct the life context of the unidentified. In this case, even without knowing when the procedure was made, or when the individual arrived in Portugal, the fact is that the scarce presence of this type of alloy in the clinical practice in Portugal would be of great value in reconstructing the individual’s identity.

## Conclusion

In forensic anthropology practice, correctly identifying a dental prosthesis with the help of a dental expert seemed a very straightforward procedure. As mentioned, restorations fabricated from Cu-Al alloys oftentimes remain similar in appearance to the gold compounds, and their misclassification is common. Hence, if an extended examination of this case had not been performed, fundamental details would be lost, and in a practical scenario, the comparison with available ante mortem data would be compromised.

The analysis of this type of device may be a fundamental step for individualization in the human identification process or to guide the expert in constructing a geographical and chronological context. Confidence in what is observed and classified is utmost. In this case, the X-ray fluorescence analysis allowed us to accurately classify the materials and contribute to knowing more about the individual’s life history. This technique is of great interest for forensic analysis due to its rapidness, non-destructive nature, and straightforward interpretation of results.

## Data Availability

Not applicable.
